# Changes in aortic reactivity associated with the loss of equilibrative nucleoside transporter 1 (ENT1) in mice

**DOI:** 10.1371/journal.pone.0207198

**Published:** 2018-11-08

**Authors:** K. Arielle Best, Derek B. Bone, Gonzalo Vilas, Robert Gros, James R. Hammond

**Affiliations:** 1 Department of Physiology and Pharmacology, Western University, London, Ontario, Canada; 2 Department of Pharmacology, University of Alberta, Edmonton, Alberta, Canada; 3 Molecular Medicine Research Group, Robarts Research Institute, London, Ontario, Canada; Max Delbruck Centrum fur Molekulare Medizin Berlin Buch, GERMANY

## Abstract

*Slc29a1* encodes for equilibrative nucleoside transporter subtype 1 (ENT1), the primary mechanism of adenosine transfer across cell membranes. Previous studies showed that tissues isolated from *Slc29a1*-null mice are relatively resistant to injury caused by vascular ischemia-reperfusion. To determine if there are similar changes in the microvasculature, and investigate underlying mechanism, we examined aortas isolated from wildtype and *Slc29a1*-null mice. Aorta macrostructure and gene expression were examined histologically and by qPCR, respectively. Wire myography was used to assess the contractile properties of isolated thoracic aortic rings and their response to adenosine under both normoxic and hypoxic conditions. In vivo haemodynamic parameters were assessed using the tail-cuff method. *Slc29a1*-null mice had significantly (P<0.05) increased plasma adenosine (2.75-fold) and lower blood pressure (~15% ↓) than wild-type mice. Aortas from *Slc29a1*-null mice were stiffer with a smaller circumference (11% ↓), and had an enhanced contractile response to KCl and receptor-mediated stimuli. Blockade of ENT1 with nitrobenzylthioinosine significantly enhanced (by ~3.5-fold) the response of aorta from wild-type mice to phenylephrine, but had minimal effect on aortas from *Slc29a1*-null mice. Adenosine enhanced phenylephrine-mediated constriction in the wild-type tissue under both normoxic (11.7-fold) and hypoxic (3.6-fold) conditions, but had no effect on the *Slc29a1*-null aortic aorta. In conclusion, aortas from *Slc29a1*-null mice respond to hypoxic insult in a manner comparable to wild-type tissues that have been pharmacologically preconditioned with adenosine. These data also support a role for ENT1 in the regulation of the protective effects of adenosine on contractile function in elastic conduit arteries such as thoracic aorta.

## Introduction

*Slc29a1* encodes for a membrane transporter with broad specificity for both purine and pyrimidine nucleosides, commonly referred to as Equilibrative Nucleoside Transporter 1 (ENT1). Adenosine, the primary endogenous substrate of ENT1 [1], is well established as a vasodilator, cardioprotectant and immunosuppressant in the cardiovasculature [2–7]. Adenosine may also play a role in tissue ‘preconditioning’ [3, 8–12], the phenomenon whereby prior exposure of tissue to ischemia-reperfusion attenuates the damage caused by subsequent ischemia-reperfusion events [13]. Enhancement of adenosine action has been explored by many investigators as a mechanism to attenuate the effects of tissue hypoxia and ischemia-reperfusion injury [4, 14–25]. ENT1 is also involved in the transport of anticancer and antiviral nucleoside analogues into cells [26], and reduction in the expression of ENT1 has been linked to the development of drug resistance is some cancers [1, 27–29].

Given the significant biological roles of adenosine and its modulation by ENT1, it is reasonable to anticipate that genetic knockout of *Slc29a1* would have obvious effects on biological function in numerous systems. Initial studies on a *Slc29a1*-null mouse [30] revealed that these ENT1-deficient animals displayed reduced anxiety in behavioural studies [31], as well as a reduced response to ethanol in spite of enhanced ethanol drinking preference [30]. Our lab has shown previously that the *Slc29a1*-null mouse develops a progressive ectopic mineralization phenotype involving the axial skeleton that resembles diffuse idiopathic skeletal hyperostosis in people [32]. We have also reported that hearts from *Slc29a1*-null mice are significantly protected from the effects of ischemia-reperfusion [16]. Others have shown a similar preconditioning-like phenotype in isolated kidneys and livers from these mice [24, 33]. These data suggest that there are adaptive changes in the *Slc29a1*-null mouse that provide a significant protective advantage to tissues under conditions of vascular stress. Understanding what these changes are may reveal previously unexplored ways to attenuate ischemia-reperfusion injury and the effects of hypoxia. While most of the known vascular actions of adenosine involve the smaller resistance vessels, adenosine can also regulate the elastic properties of conduit arteries, such as the thoracic aorta, via adenosine receptor-mediated nitric oxide production [34, 35]. In the present study, we show that loss of ENT1 in the *Slc29a1*-null mice results in significant changes in the contractile activity of the thoracic aorta and its response to hypoxic insult.

## Materials and methods

### Reagents

All reagents were purchased from Sigma-Aldrich (Oakville, Ontario, Canada), with the exception of the following. The oligonucleotide primers for PCR were prepared by Integrated DNA Technologies (Iowa, USA). Hematoxylin and DNA polymerase were purchased from ThermoFisher Scientific Canada (Burlington, Ontario, Canada), and SCH58261 was from Tocris Bioscience (via Cederlane Corp., Burlington, Ontario, Canada).

### Animals

*Slc29a1*-null mice were obtained originally from Dr Doo-Sup Choi (Mayo Clinic, Rochester, MI, USA) and used to establish a breeding colony at the University of Western Ontario (London, Canada), and subsequently at the University of Alberta (Edmonton, Canada). The *Slc29a1*-null mice were bred with C57BL/6 mice as described previously by Chen et *al*. (2007) [31]. Maintenance of the mouse colony occurred through the breeding of *Slc29a1*^+/-^ (heterozygous) pairs to obtain *Slc29a1*^+/+^ (Wild-type) and *Slc29a1*^-/-^ (*Slc29a1*-null) littermates. All procedures were in accordance with policies and guidelines outlined by the Canadian Council on Animal Care and approved by the Animal Use Subcommittees of the University of Western Ontario and the University of Alberta. These Canadian policies and guidelines comply with National Institutes of Health guide for the care and use of Laboratory animals (NIH Publications No. 8023, revised 1978). Mice were housed under a 12 h light/dark cycle and allowed unlimited access to rodent chow and drinking water. Male mice were utilized for this study to reduce the influences of hormonal cycles on the vascular functions being studied. Previous studies using the *Slc29a1*-null mouse showed no obvious gross anatomical abnormalities up to the age of about 4 months. Mice older than 4 months showed spine stiffness and gait disturbances [32, 36], likely due to the progressive ectopic mineralization that has been reported in these mice [32, 37]. Therefore, the data reported herein are from mice of 3–4 months of age. In addition to litter genotyping, loss of ENT1 protein in the *Slc29a1*-null mice was confirmed periodically (every 3–4 months) by the lack of high affinity binding of the ENT1-selective high affinity probe [^3^H]nitrobenzylthioinosine (NBMPR).

### Plasma adenosine

Mice were anaesthetized with pentobarbital and blood was collected by cardiac puncture into a 1 ml syringe preloaded with 0.2 ml of a ‘stop’ solution composed of 118 mM NaCl, 5 mM KCl, 13.2 mM EDTA, 10 μM 5-iodotubercidin (adenosine kinase inhibitor), 100 μM erythro-9-(2-Hydroxy-3-nonyl)adenine hydrochloride (EHNA; adenosine deaminase inhibitor), and 10 μM dilazep (ENT inhibitor) [38]. Sampled blood was immediately mixed with the stop solution by inverting the syringe several times; this mixture was designed to prevent metabolism of adenosine during subsequent sample processing. Serum was isolated by centrifugation at 3000 x g for 10 min at 4°C and then applied to a 10 kDa cut-off ultra-filtration column and centrifuged at 14 000 x g for 15 min at 4°C. Filtrate was used for HPLC analysis using an Onxy monolithic C18 column with a slightly modified protocol as established by Farthing *et al* [39] where adenosine was detected with a UV detector at 260 nm and the adenosine metabolites xanthine and uric acid were detected at 250 nm.

### Haemodynamics

Due to the reported anxiolytic phenotype of the *Slc29a1*-null mice [31], all haemodynamic parameters were derived using anaesthetized animals to eliminate behavioral influences on sympathetic neuronal regulation of vascular function. Heart rate and blood pressure were determined using the CODA-6 non-invasive tail-cuff method (Kent Scientific Corporation, Torrington, CT, USA). Anaesthetized animals (1.5% isoflurane in O_2_) were kept on a heated pad to prevent loss of body temperature. Animals were subjected to five acclimatization rounds (no data collection) followed by two separate acquisition cycles of 15 measurements each. There was a 60 s rest period between acquisition cycles. The tail cuff was deflated over a period of 20 s during data acquisition.

### Gene expression

Thoracic aortas were frozen in liquid nitrogen immediately upon dissection. Frozen tissue was placed in a microcentrifuge tube to which 0.5 ml Trizol was added along with a stainless steel bead and homogenized using a TissueLyser (Qiagen, Toronto, ON, Canada). Once complete tissue homogenization was obtained (~5 min), 0.1 ml of chloroform was added, vortex mixed, and then centrifuged for 15 min at 12,000 x *g* at 4°C. The top aqueous layer was transferred to a new microcentrifuge tube, and centrifuged again for 15 min at 12,000 *x g* at 4°C. The aqueous layer from this tube (~200 μl) was placed in a fresh microcentrifuge tube and an equal volume of 70% ethanol added. Total RNA was extracted using a Qiagen RNesy Mini Kit with a 30 μl final elution volume using procedures specified by the kit manufacturer. All samples were subjected to in-column treatment with DNase I (Qiagen, Toronto, ON, Canada). RNA concentration and quality was assessed using a NanoDrop 2000 spectrophotometer (Life Technologies Inc., Burlington, ON, Canada), and then stored -80°C. cDNA was generated from 0.5 μg of RNA using SuperScript II Reverse Transcriptase and Oligo(dT) 12–18 primers (Invitrogen). Real-time PCR was carried out in a StepOnePlus instrument (Applied Biosystems, Life Technologies, Burlington, ON, Canada) using Power SYBR Green PCR Master Mix (Invitrogen) and primers designed, and verified, to amplify the transcripts as shown in S1 Table. Mouse *UBC*, *eIF4a2*, and *ActB* were used as reference genes and their geometric mean was used to calculate a normalization index [40]. Target gene expression was calculated using the relative standard curve method.

### Histology

Mice were anesthetized with ketamine/xylazine and perfused via the left ventricle with phosphate-buffered saline (PBS) and then paraformaldehyde (4% wt/vol) under physiological pressure for 30–45 min [41]. After immersion in 4% paraformaldehyde for overnight, two-mm segments of the thoracic aorta were embedded in paraffin and cut into 5 μm-thick serial sections. The tissues were sectioned in the coronal plane, mounted on glass slides and baked at 45°C for 48 h. Samples were then stained with hematoxylin and eosin (H&E) or Alizarin Red (to detect calcification) counterstained with H&E. Sections were subsequently dewaxed in xylene and rehydrated via consecutive immersion in decreasing concentrations of ethanol to aid in the visualization of the cell nuclei. Shur/Mount xylene-based liquid mounting media (Durham, NC, USA) was used.

### Aortic ring myography

Vascular reactivity was assessed in aortic rings in accordance with previously published methods [42, 43]. A DMT (Danish Myo Technology) Myograph 620M was used to measure the tension generated, with data rendered using a PowerLab 4/30 (ADInstruments) and LabChart 7 Pro software (ADInstruments, Australia). Mice were anaesthetized with pentobarbital sodium (540 mg/kg ip), the chest wall was opened and the thoracic aorta removed and transferred to a Petri dish (on ice) containing Krebs physiological salt solution (KPSS; composition (mmol/l)—NaCl 118.0, NaHCO_3_ 25.0, d-glucose 11.1, KCl 4.72, CaCl_2_2H_2_O 2.56, NaH_2_PO_4_2H_2_O 1.13, MgCl_2_6H_2_O 1.12, (-) ascorbic acid 0.114, and disodium EDTA 0.03). Connective tissue and blood were removed and four ring segments (2 mm in length) from each aorta were cut and mounted in organ baths individually gassed with 95% O_2_ and 5% CO_2_ and maintained at 37°C. The length-tension relationship of the thoracic aortas was assessed following a 30 min equilibration period by increasing the stretch of the tissue by 50 microns every 2 min until the tension generated reach a maximum. Subsequent experiments were done under an applied tension of 0.5 g, to simulate physiological pressure. The rings were equilibrated for 30 min before being constricted with 100 mM KCl to assess tissue viability. To assess receptor-mediated contractility, aortic rings were exposed to increasing doses of phenylephrine (1 nM– 30 μM), prostaglandin F2α (PGF2α; 10 nM– 30 μM) or 5-hydroxytryptamine (5-HT; 1 nM– 30 μM). In some cases, tissue was treated with NBMPR; 1 μM) or dimethyl sulfoxide (DMSO; 0.1%) for 15 min prior to application of constricting agent. To assess vasodilatory responses to adenosine, aortic rings were pre-constricted with PGF2α (80% of maximum using a predetermined concentration) and then exposed to increasing adenosine concentrations at two min intervals. KPSS in the organ bath was changed three times after each treatment.

### Effect of hypoxia

Aortic rings were exposed to hypoxic (95% N_2_−5% CO_2_) or normoxic (95% O_2_−5% CO_2_) conditions for 60 min, rinsed three times with KPSS, and returned to normoxic conditions for an additional 30 min. To assess the effect of blockade of ENT1 on the response of the tissue to hypoxia, aortas were exposed to NBMPR (1 μM) for 15 min prior to the normoxic/hypoxic period and maintained at that concentration throughout the 60 min of hypoxia. To examine the ability of adenosine to precondition the tissue, aortas were exposed to adenosine (10 μM) for 30 min prior to the initiation of hypoxia (or normoxia control). Aortic rings were then exposed to increasing doses of phenylephrine (1 nM– 30 μM), with results expressed as a percentage of the initial KCl-induced maximum contraction (determined prior to any treatment).

### Statistical analysis

Results are reported as mean ± SEM from at least 5 independent experiments (N values are shown in figure legends). Data were analyzed using non-linear regression and statistical analyses were performed using Prism 7.02 (GraphPad Software Inc., San Diego, CA). Data sets were compared using a two-tailed Student’s *t*-test or a 2-way ANOVA with Bonferroni post-tests as appropriate. Significance was determined based on a P value of 0.05.

## Results

### Plasma adenosine levels

**Fig 1:**
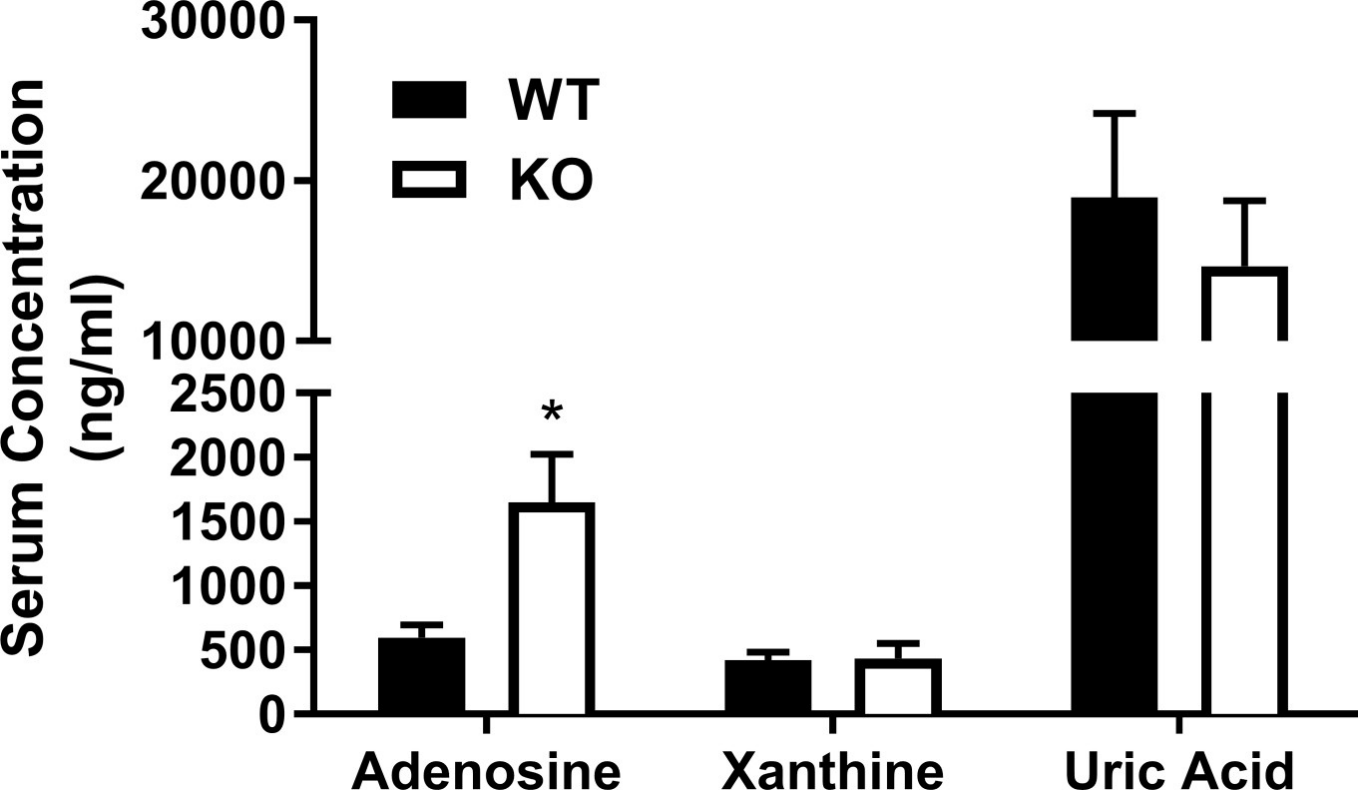
Plasma adenosine levels. Blood was obtained from wild-type (WT) and *Slc29a1*-null (KO) mice via cardiac puncture (in the presence of dilazep, EHNA and iodotubercidin to limit adenosine metabolism) and processed for HPLC analysis of adenosine, xanthine and uric acid content. *Significant difference between wild-type and *Slc29a1*-null mice for the indicated parameter (Student’s t-test for unpaired samples, two-tailed, P<0.05, N = 5).

Plasma adenosine levels were significantly elevated (2.75-fold) in *Slc29a1*-null mice (1650 ± 370 ng/ml) compared to wild-type mice (600 ± 100 ng/ml). There were no differences in xanthine or uric acid concentrations between genotypes (Fig 1).

### Haemodynamics

**Fig 2:**
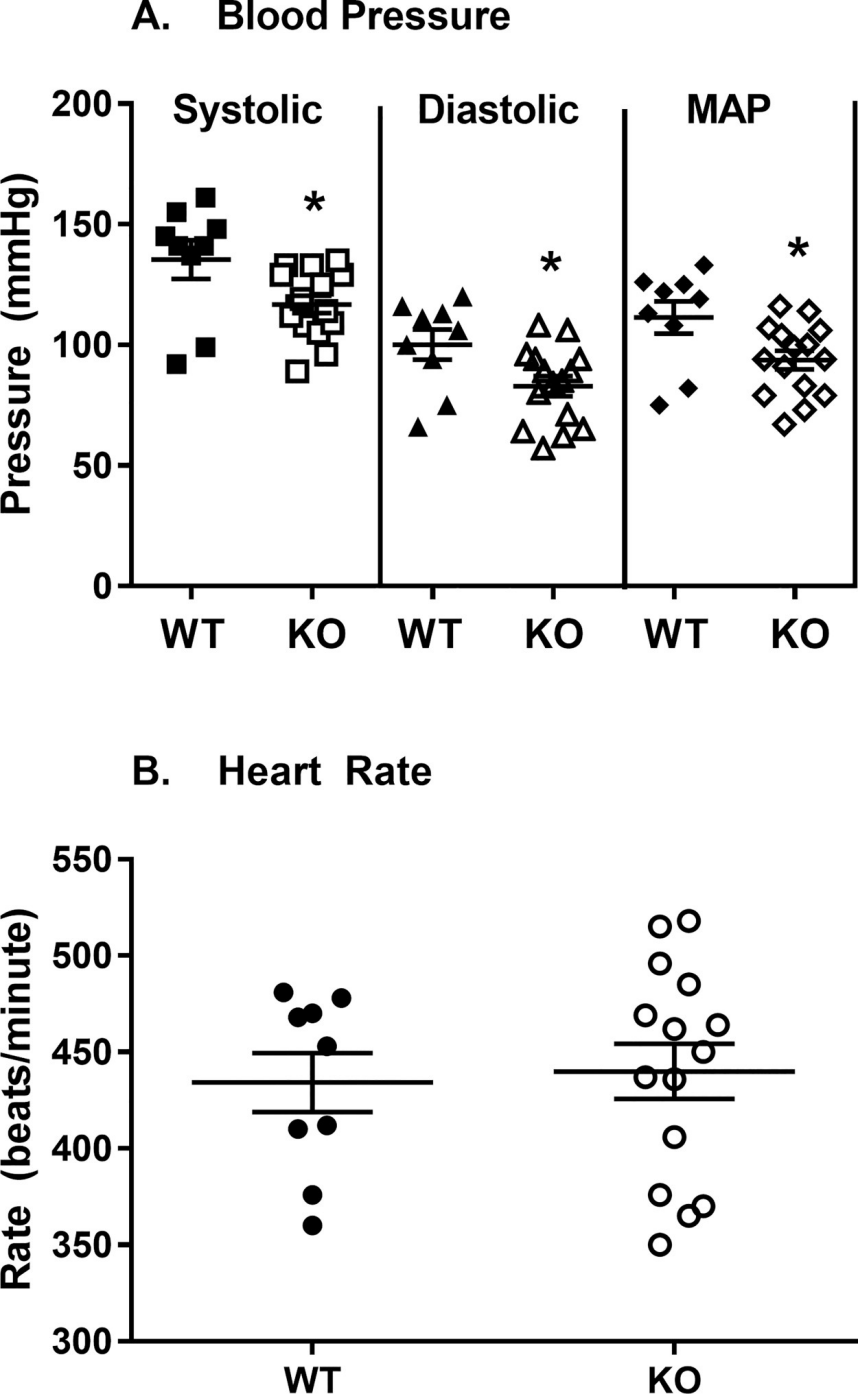
Haemodynamic measurements. Systolic, diastolic, mean arterial pressure (MAP) (Panel A), and heart rate (Panel B), were measured in anesthetized wild-type (WT) and *Slc29a1*-null (KO) mice using the tail-cuff method. *Significant difference between wild-type and *Slc29a1*-null mice for the indicated parameter (Student’s t-test for unpaired samples, two-tailed, P<0.05).

*Slc29a1*-null mice had significantly lower systolic (117 ± 4 mmHg) and diastolic (83 ± 4 mmHg) blood pressure than wild-type mice (135 ± 8 mmHg and 100 ± 6 mmHg for systolic and diastolic pressure, respectively) (Fig 2A). This change occurred in the absence of any difference in heart rate between the wild-type (434 ± 15 bpm) and *Slc29a1*-null (439 ± 15 bpm) mice (Fig 2B).

### Histology

**Fig 3:**
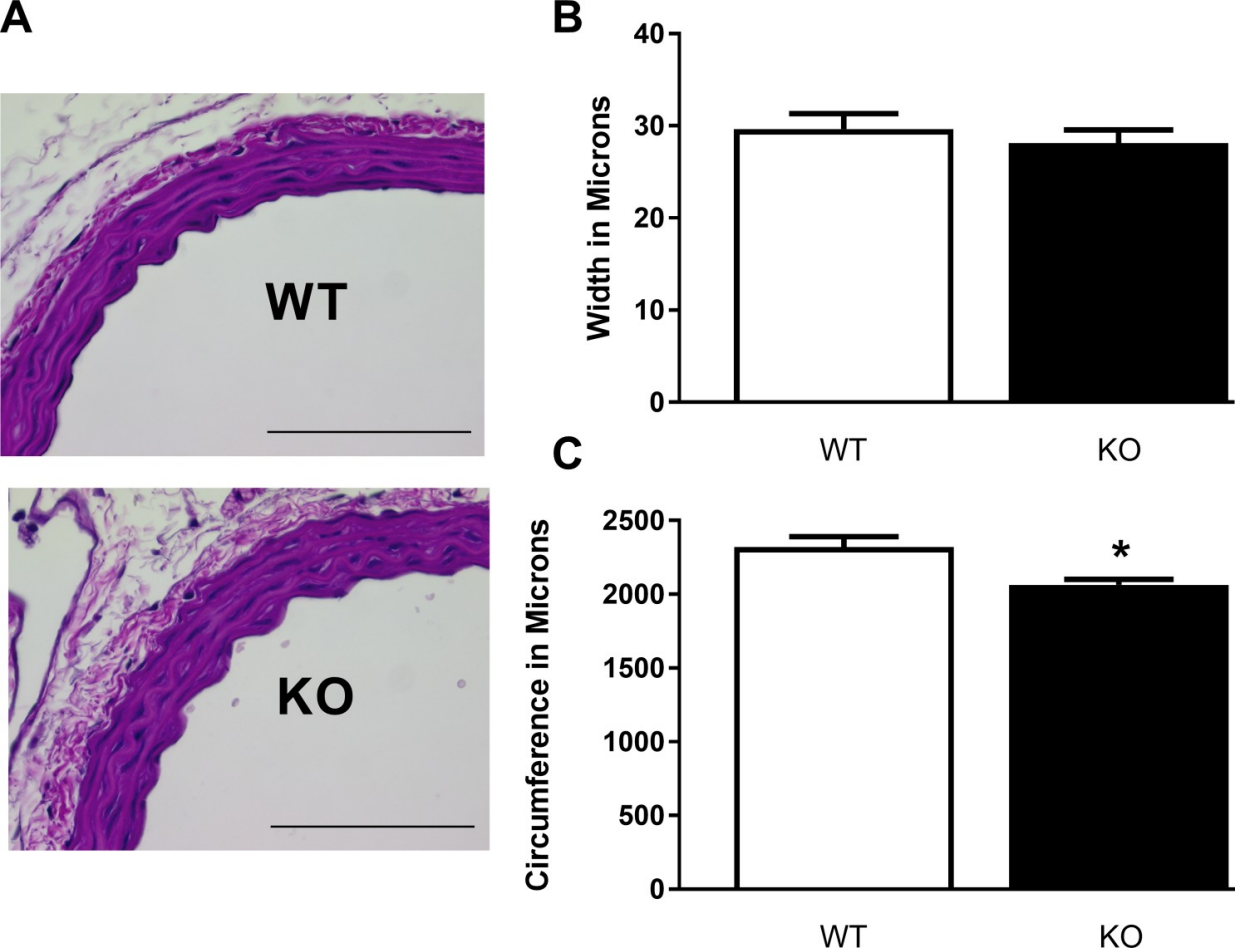
Histological appearance of thoracic aorta in wild-type (WT) and Slc29a1-null (KO) mice. Tissues were gravity perfusion fixed with paraformaldehyde, sectioned in the transverse plane and stained with haematoxylin and eosin. Layers of elastin as well as nuclei arrangement were analyzed (Panel A). Images are representative of six animals of each genotype. Scale bars represent 100 μm. Panel B shows the vascular wall thickness and lumen circumference derived from these tissue samples (Mean ± SEM, N = 6). * Significant difference in measured parameter between the wild-type and *Slc29a1*-null samples (Student’s t-test for unpaired samples, P<0.05).

Histological examination of the thoracic aorta showed normal vessel morphology in the *Slc29a1*-null mice (Fig 3A) with no difference in the width of the vessel wall compared with wild-type (Fig 3B). However, aortas from the *Slc29a1*-null mice were significantly smaller in circumference (2064 ± 36 versus 2320 ± 71 microns for aortas from the *Slc29a1*-null and wild-type mice, respectively; Fig 3C).

### Gene expression

**Fig 4:**
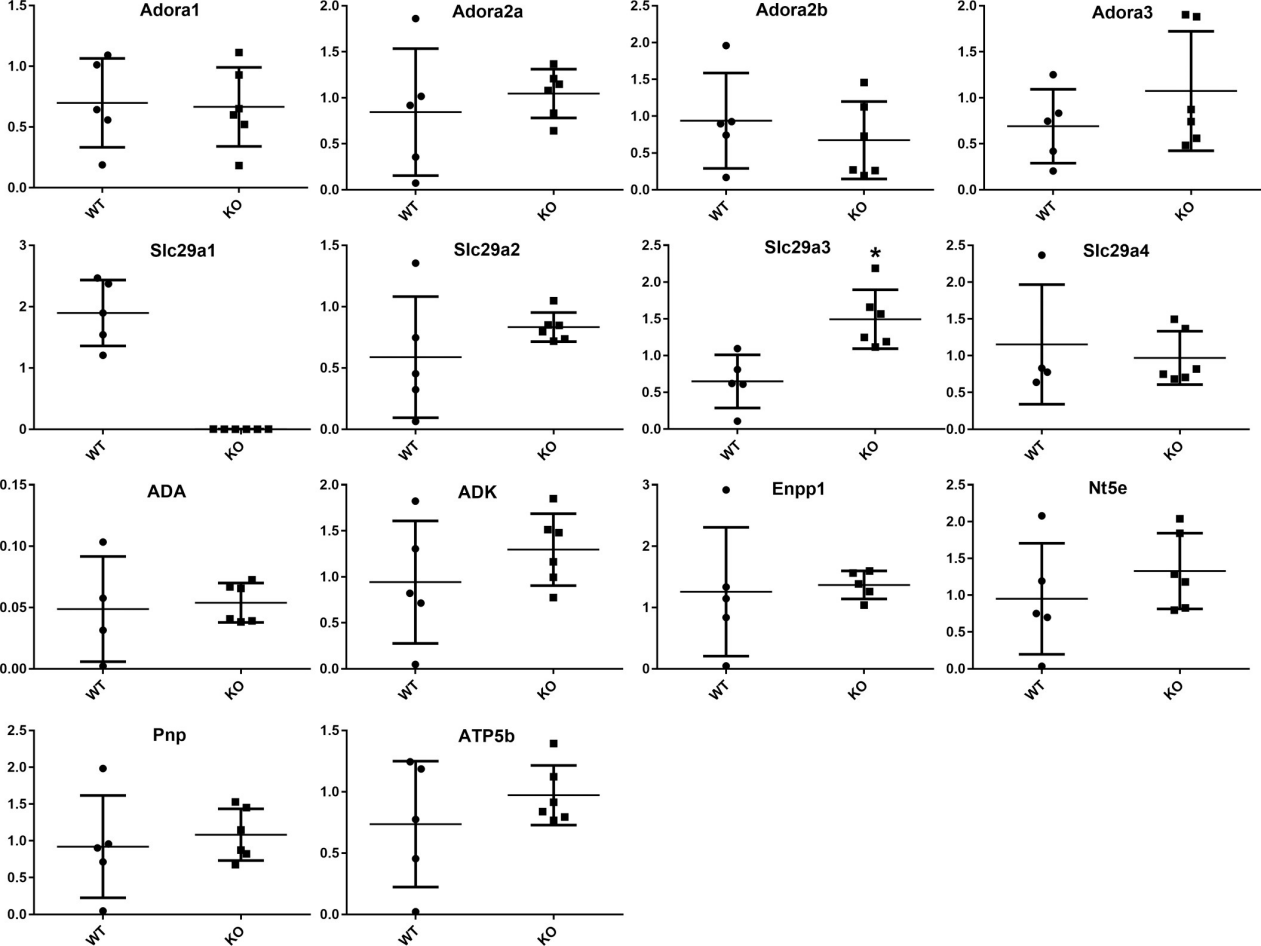
Gene expression in wildtype and Slc29a1-null mice: qPCR was employed, using the primer sets shown in S1 Table, to assess the relative expression of the equilibrative nucleoside transporters, *Slc29a1*, *Slc29a2*, *Slc29a3*, and *Slc29a4*, the adenosine receptors, *Adora1*, *Adora2a*, *Adora2b*, and *Adora3*, and enzymes involved in purine metabolism, *ADA*, *Nt5e*, *ADK*, *Pnp*, *Enpp1*, and *ATP5b*. Gene expression (mean ± SEM) is shown as relative to the geometric mean of the reference genes *UBC*, *eIF4a2*, and *ActB*. * Significant difference between wild-type (N = 5) and *Slc29a1*-null (N = 8) mice (Student’s t-test for unpaired samples, two-tailed, P<0.05).

A consequence of global knockout of *Slc29a1* may be compensatory changes in the expression of other components of the purinergic regulatory system. Real-time semi-quantitative PCR revealed no change in the expression of genes encoding other plasma membrane-located equilibrative nucleoside transporters (*Slc29a2* or *Slc29a4*) in the thoracic aorta. Nor was there a difference in expression of any of the adenosine receptors (*Adora1*, *2a*, *2b*, *3*), or adenosine metabolic enzymes (*Ada*, *Adk*, *Enpp1*, *Nt5e*, *Pnp*, *ATP5b*). The only significant difference observed was a 2.3-fold increase in transcript (*Slc29a3)* for the intracellular lysosomal-located equilibrative nucleoside transporter subtype 3 (ENT3) in the aortas from *Slc29a1*-null mice (Fig 4).

### Vascular reactivity

**Fig 5:**
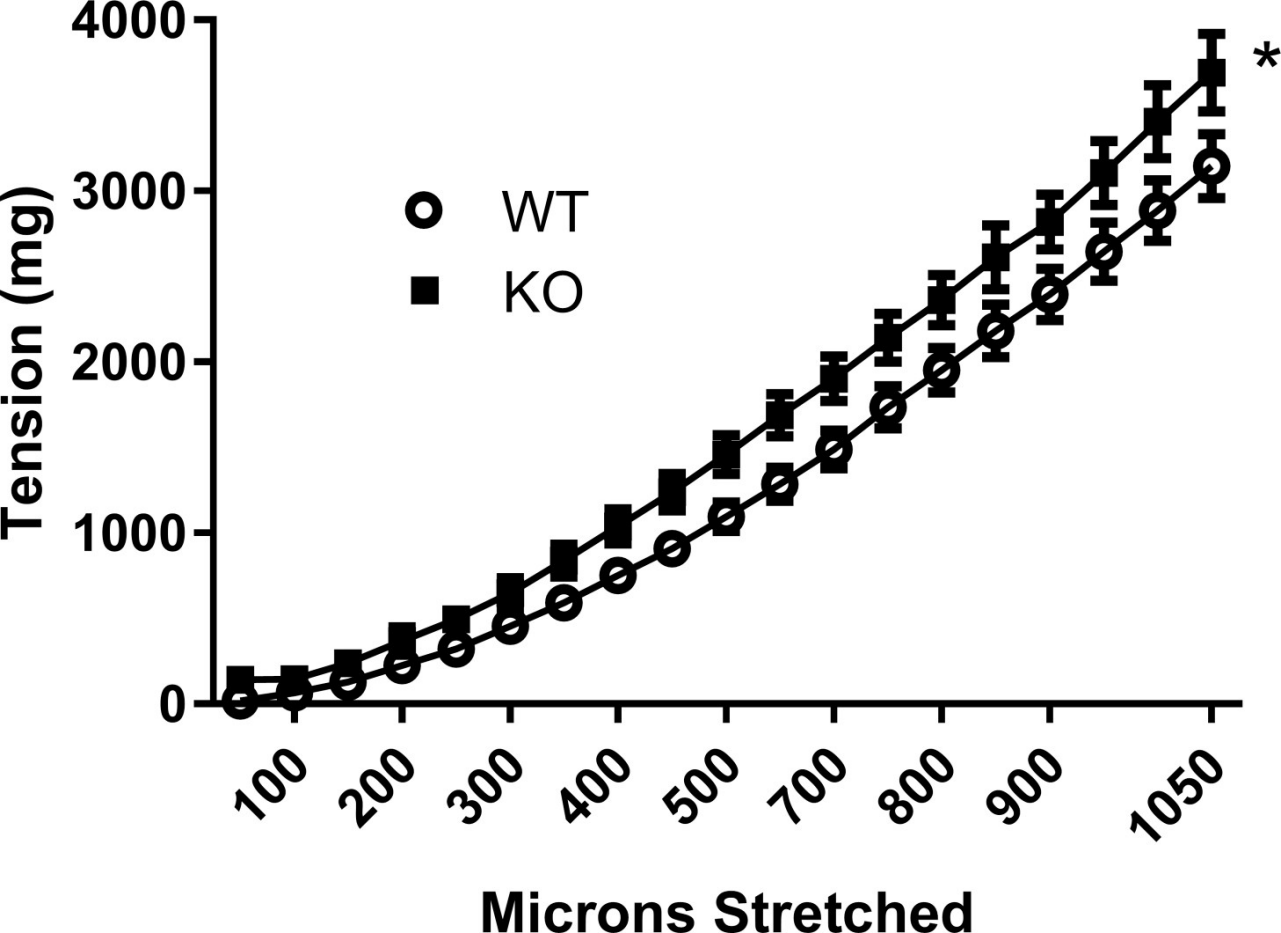
Length-tension relationship for aorta from wild-type (WT) and Slc29a1-null (KO) mice. Tissues were incubated for 30 min at 22°C in KPSS buffer with no resting tension, prior to increasing tension in 50 micron increments. Each point represents the mean ± SEM, N = 7. *Significant difference between aortas from wild-type and *Slc29a1*-null mice (Two-Way ANOVA for unpaired experimental parameters, P<0.05).

**Fig 6:**
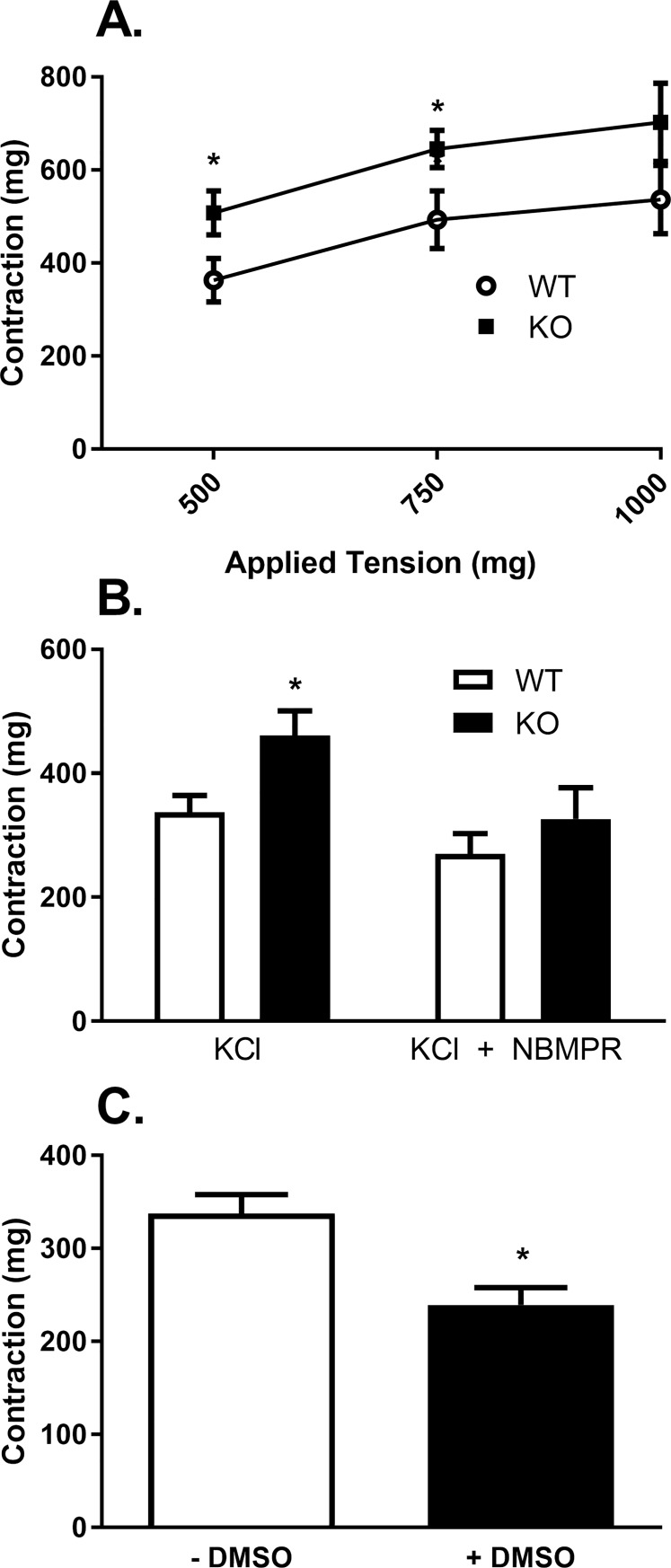
KCl-induced contraction of thoracic aorta from wild-type (WT) and Slc29a1-null (KO) mice. Tissues were incubated for 30 min at 37°C in KPSS buffer at resting tensions of 500 mg, 750 mg and 1000 mg. Following equilibration period, 100 mM KCl was administered to assess tissue viability (Panel A). Each point represents the mean ± SEM, N = 6. Panel B shows the effect of NBMPR on KCl-induced contractions of the thoracic aorta from wild-type and *Slc29a1*-null mice. Tissues were incubated for 30 min at 37°C in KPSS buffer at resting tensions of 500mg in the absence and presence of 1 μM NBMPR, and then 100 mM KCl was administered. Each bar represents the mean ± SEM, N = 7. NBMPR was prepared in DMSO with a final concentration of DMSO in the tissue bath of 0.1% v/v. Panel C shows the effect of 0.1% DMSO alone on the KCl-induced contraction of wild-type thoracic aortas (N = 5). *Significant difference between aortas from wild-type and *Slc29a1*-null mice (Panel A and B) or ± DMSO (Panel C) (Student’s t-test, * P<0.05).

Aortic rings from *Slc29a1*-null mice had a significantly increased length-tension profile (Fig 5) compared with aortas from wild-type mice. Aortic rings from *Slc29a1*-null animals also had a significantly increased contractile response to KCl over a range of applied tensions (Fig 6A). To determine whether this enhanced KCl contraction was due to the loss of ENT1 function, aortas from both wild-type and *Slc29a1*-null mice were incubated with the ENT1-selective blocker NBMPR (1 μM, supramaximal inhibitory concentration). NBMPR eliminated the difference in KCl-induced contraction between the aortas from wild-type and *Slc29a1*-null mice (Fig 6B) suggesting that the difference was indeed due to the loss of ENT1. However, the overall response to KCl in the presence of NBMPR was lower relative to the controls in the aortic rings from both wild-type and *Slc29a1*-null mice. When the KCl contraction of aorta from wild-type mice was measured with and without incubation with 0.1% v/v DMSO (the solvent for NBMPR), it was found that DMSO itself caused about a 30% reduction in the response of the aorta to 100 mM KCl (Fig 6C).

**Fig 7:**
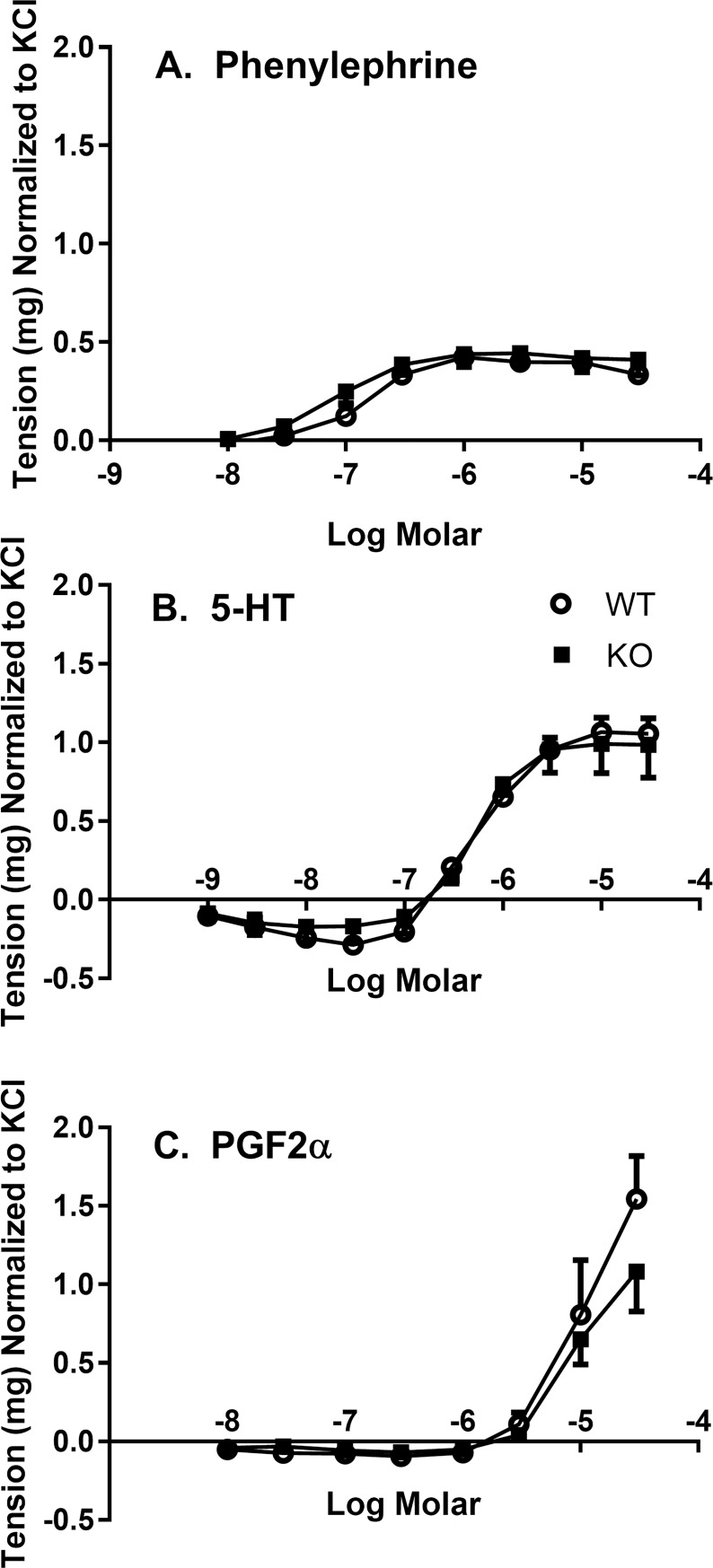
Dose-response curves for receptor-mediated contraction of thoracic aortas isolated from wild-type (WT) and Slc29a1-null (KO) mice. Tissues were incubated for 30 min at 37°C in KPSS buffer at resting tensions of 500 mg after which they were depolarized with 100 mM KCl, washed thrice, and then exposed to increasing concentrations of phenylephrine (Panel **A**), 5-HT (Panel **B**) or PGF2α (Panel **C**). Each point represents the mean ± SEM from 5 independent experiments, and expressed as tension generated normalized to the 100 mM KCl-induced contraction. No significant differences were observed between aortas from wild-type and *Slc29a1*-null mice in any of the data sets shown (Two-Way ANOVA for paired experimental parameters, P<0.05).

Receptor-mediated contraction of isolated thoracic aorta by 5-HT, phenylephrine, or PGF2α was not different between the tissues from wild-type and *Slc29a1*-null mice when normalized to the KCl-induced contractions (Fig 7). 5-HT produced a maximum tension of 1.06 ± 0.06 mg and a logEC_50_ of -6.25 ± 0.08 in the aorta from wild-type mice, whereas aorta from *Slc29a1*-null mice showed a maximum tension of 0.99 ± 0.07 mg and a logEC_50_ of -6.28 ± 0.10. Phenylephrine produced a maximum tension of 0.39 ± 0.02 mg with a logEC_50_ of –6.88 ± 0.08 in the aorta from wild-type mice, and a maximum tension of 0.43 ± 0.03 mg with a logEC_50_ of -7.10 ± 0.19 in the aorta from *Slc29a1*-null mice.

**Fig 8:**
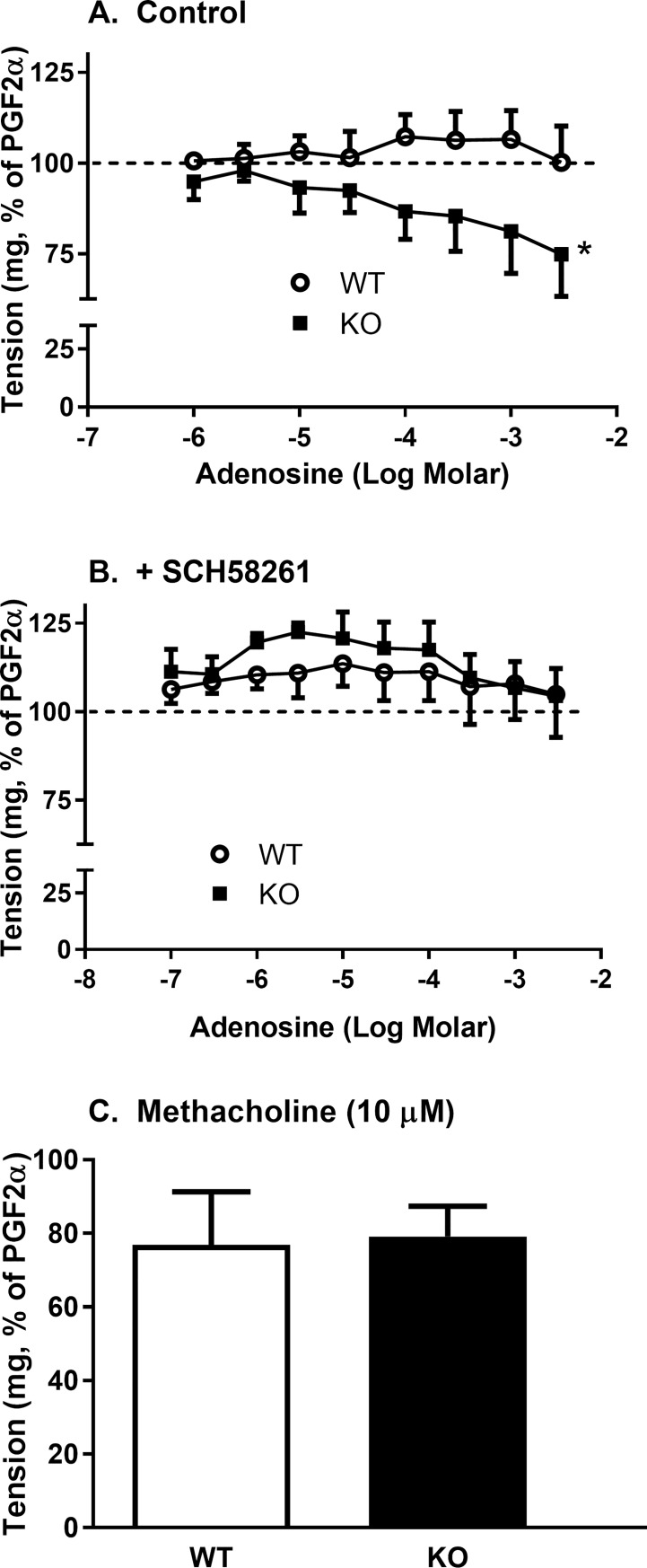
Adenosine induced vasodilation of thoracic aortas isolated from wild-type (WT) and Slc29a1-null (KO) mice. Tissues were incubated for 30 min at 37°C in KPSS buffer at resting tensions of 500 mg after which they were depolarized with 100 mM KCl to test for viability. Aortic rings were pre-constricted with PGF2α to 80% of maximum (dose range 1 μM– 10 μM) and then exposed to increasing concentrations of adenosine at two min intervals, in the absence (Control; Panel A) or presence (Panel B) of the adenosine A2 receptor blocker SCH58261 (100 nM), or 10 μM methacholine (Panel C). Relaxation responses were expressed as a percentage of the initial PGF2α contraction (shown by a dashed line in Panels A and B). Each point represents the mean ± SEM from 5 experiments. *Significant difference in response between aortic rings from wild-type and *Slc29a1*-null mice (Two-Way ANOVA for unpaired experimental parameters, *P<0.05).

When adenosine was applied to aortas pre-constricted with PGF2α there was no adenosine-induced relaxation observed in aortas from wild-type mice at any concentration tested (up to 3 mM), but there was a significant dose-dependent relaxation of aortic rings from *Slc29a1*-null mice (Fig 8A). The effect of adenosine on aortas from *Slc29a1*-null mice was prevented by the adenosine A2 receptor blocker SCH58261 (100 nM) (Fig 8B). SCH58261 alone also significantly increased the PGF2α-mediated constriction, particularly in the aortic rings from *Slc29a1*-null mice (Fig 8B). Aortas from both wild-type and *Slc29a1*-null mice, pre-constricted with PGF2α, showed a similar relaxant response to 10 μM methacholine (~20% decrease in tension) (Fig 8C), confirming that the aortas had a viable endothelium, and that loss of ENT1 activity did not affect vascular endothelial function in this model.

### Response to hypoxia

**Fig 9:**
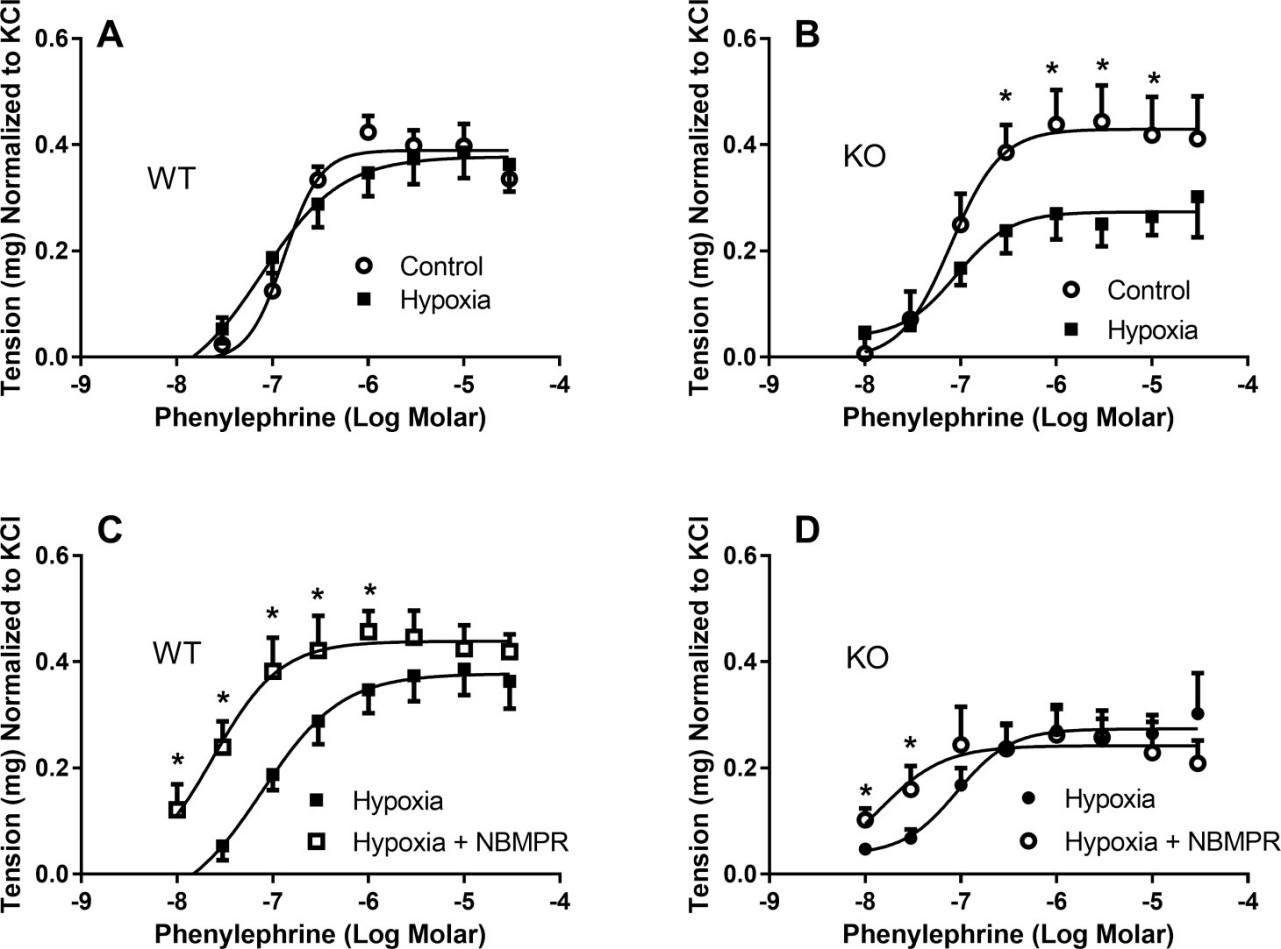
Phenylephrine dose-response curves for isolated thoracic aorta from wild-type (WT) and Slc29a1-null (KO) mice after exposure to hypoxia. Tissues were incubated for 30 min at 37˚C in KPSS buffer at resting tensions of 500 mg after which they were depolarized with 100 mM KCl. Tissues were then incubated for 60 min under either normoxic or hypoxic conditions, and then exposed to increasing concentrations of phenylephrine (Panels A and B). Panels C (WT) and D (KO) show the effect of 1 μM NBMPR on the response to hypoxia. NBMPR was administered 15 min prior to hypoxia, and then removed prior to the construction of the phenylephrine dose-response relationship. Each point is the mean ± SEM from 6 (Panel A & B) or 5 (Panel C and D) experiments. *Significant difference between treatment groups (Student’s t-test for unpaired samples, *P<0.05).

Sixty min of hypoxia had no effect on the response of aortas from wild-type mice to phenylephrine (Fig 9A). Aortas from *Slc29a1*-null mice, on the other hand, had a significantly lower contractile response to phenylephrine after hypoxic insult relative to the normoxic controls (maximum tension of 0.27 ± 0.02 mg and 0.43 ± 0.03 mg in hypoxic versus normoxic conditions, respectively) (Fig 9B). Pre-incubation of the aortic rings with NBMPR increased the maximum tone produced by phenylephrine in the aortas from wild-type mice (maximum tension of 0.44 ± 0.01 mg versus 0.37 ± 0.03 mg in the presence and absence of NBMPR) (Fig 9C), but had no effect on the maximum tone generated in aortas from *Slc29a1*-null mice (Fig 9D). However, NBMPR enhanced the apparent potency of phenylephrine, under hypoxic conditions, in aortas isolated from both wild-type (logEC_50_ = -7.62 ± 0.04 and -7.11 ± 0.26 in the presence and absence, respectively, of NBMPR) and *Slc29a1*-null mice (logEC_50_ of -7.85 ± 0.11 and -7.04 ± 0.25 in the presence and absence, respectively, of NBMPR).

**Fig 10:**
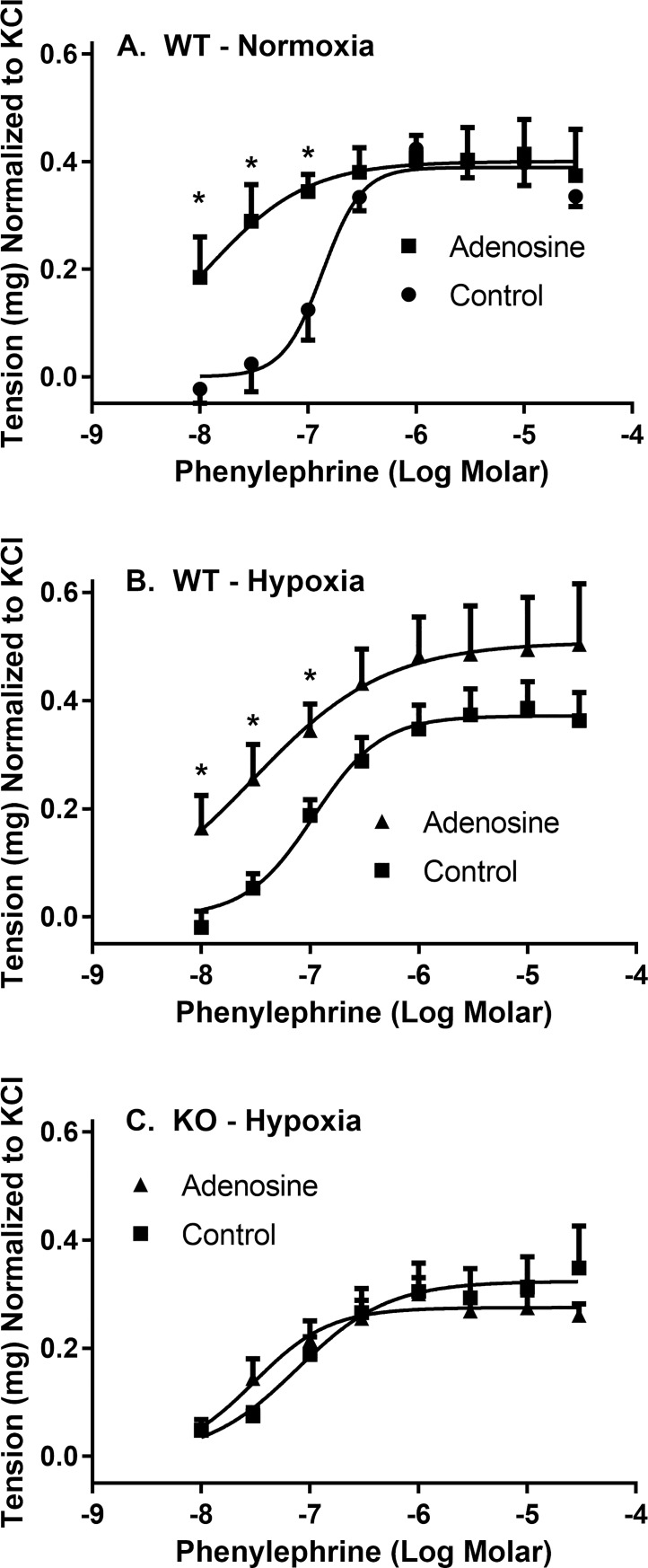
Effect of adenosine pre-incubation on phenylephrine-induced contractions of isolated thoracic aorta from wild-type (WT) and Slc29a1-null (KO) mice. Tissues were incubated for 30 min at 37°C in KPSS buffer at resting tensions of 500 mg after which they were depolarized with 100 mM KCl. Tissues were then incubated in the presence or absence of 10 μM adenosine for 30 min, and then washed and incubated for an additional 60 min under either normoxic (Panel A) or hypoxic (Panels B and C) conditions. Each point is the mean ± SEM from 6 experiments. *Significant difference between treatment groups (Student’s t-test for unpaired samples, *P<0.05).

Pre-incubation of aorta from wild-type mice with 10 μM adenosine also enhanced the potency (by ~12-fold) of phenylephrine in the generation of vessel tone (logEC_50_ of -7.94 ± 0.22 and -6.87 ± 0.08 with and without adenosine, respectively), with no change in the maximum contraction produced (Fig 10A). Under hypoxic conditions in aortas from wild-type mice, adenosine increased the potency of phenylephrine by 3.6-fold (logEC_50_ of -7.53 ± 0.26 and -6.97 ± 0.12 with and without adenosine, respectively), and also enhanced the maximum contraction achieved (0.51 ± 0.05 and 037 ± 0.02 mg tension with and without adenosine, respectively) (Fig 10B). These effects of adenosine were absent in the aortas from *Slc29a1*-null mice under both normoxic and hypoxic conditions (Fig 10C).

## Discussion

The primary goal of this study was to examine the characteristics of the isolated thoracic aorta from wild-type and *Slc29a1*-null mice under hypoxic and normoxic conditions. In conjunction with this analysis, the *in vivo* haemodynamic parameters and plasma adenosine levels in these mice were assessed. Given the important role of adenosine in regulating vascular function, the loss of ENT1 in the *Slc29a1*-null mice may be expected to have a significant impact on response of vascular tissue to hypoxia and the preconditioning effect of adenosine.

### Plasma adenosine levels and *in vivo* haemodynamics

The plasma concentration of adenosine was elevated by almost 3-fold in the *Slc29a1*-null mice relative to wild-type mice. Increased plasma adenosine levels in the *Slc29a1*-null mice has also been reported by others [44], as has increased plasma thymidine levels [45]. These data are compatible with the concept that the adenosine gradient across the plasma membrane is inwards under physiological conditions [46, 47], where loss of ENT1 would prevent accumulation of adenosine by tissues, and its subsequent metabolism by intracellular adenosine kinase and adenosine deaminase, leading to an increase in circulating adenosine concentrations. Anaesthetized *Slc29a1*-null mice also had decreased blood pressure relative to wild-type mice, which is congruous with the increased circulating adenosine levels leading to enhanced vasodilatory tone. Interestingly, this also suggests minimal compensation in other adenosine signalling and metabolic components in response to the loss of ENT1 activity. This is supported by our findings that there are no significant differences in the expression of transcripts for adenosine receptors, adenosine metabolizing enzymes or other plasma membrane nucleoside transporter subtypes in aortas from wild-type and *Slc29a1*-null mice. A previous study also showed that hearts isolated from wild-type and *Slc29a1*-null mice do not differ in the expression of these genes [16]. It remains possible that there were changes in gene expression and/or enzyme activities in other tissues that may affect adenosine metabolism in the *Slc29a1*-null mice. Nevertheless, regardless of underlying mechanism, the end result was an increase in circulating adenosine.

### Thoracic aorta contractility

The thoracic aorta is an elastic artery that plays a crucial role in damping the pressure wave that occurs due to the pulsatile nature of cardiac function, to maintain a relatively constant pressure of flow to the circulatory system. Thus, changes in aortic reactivity can have significant effects on cardiac function and tissue perfusion. Aorta from *Slc29a1*-null mice were slightly constricted (reduced lumen circumference) and stiffer (increased length tension relationship) than aorta from wild-type mice. The reduced lumen size may reflect a compensation to the increased vasodilatory tone in these animals [48]. Increased aorta stiffness is normally associated with hypertension and ageing, and is thus a paradoxical finding in this study where there was actually a decreased blood pressure in these relatively young (3-month) *Slc29a1*-null animals. Histochemical analysis revealed no changes in vascular wall organization, nor in the elastic fiber layers. Previous microCT imaging studies revealed no abnormal mineralization of the aorta from *Slc29a1*-null mice [32], and we found no evidence of mineralization of these tissues based on Alizarin Red staining. However, we cannot rule out changes in collagen content. Increased signalling via adenosine A2a receptors has been shown to increase collagen synthesis [49], and the mouse aorta does express A2a receptors. While we did not see any change in A2a receptor expression in aortas from *Slc29a1*-null mice (relative to wild-type), the enhanced circulating adenosine levels may result in increased A2a receptor signalling. The enhanced contraction induced by KCl-depolarization in the aortas from *Slc29a1*-null mice may be a reflection of this increased vessel stiffness and decreased circumference. There is a complex interplay between vessel diameter, preload, and stiffness that can impact tissue biomechanics [50]. The increased contraction of the *Slc29a1* aortic rings may also be due to the inability of adenosine to be released, via ENT1, from the contracting muscle cells, thereby attenuating a normal compensatory adenosine-mediated vasodilation. Our finding that the ENT1 blocker, NBMPR, eliminated the difference in KCl-induced contraction between the aortas from wild-type and *Slc29a1*-null mice would support this hypothesis. It is noteworthy that DMSO itself (the solvent for NBMPR) had a significant effect on vascular contractility in this study. A similar vasodilatory effect of DMSO has been reported for rat aorta [51]. Pre-incubation of aortic rings isolated from wild-type mice with NBMPR lead to an enhancement in the maximum tone generated by phenylephrine. This effect of NBMPR was not seen in aortic rings from *Slc29a1*-null mice, suggesting that it was a consequence of ENT1 blockade. However, NBMPR pre-incubation also had a significant enhancing effect on the potency of phenylephrine in aortic rings from both wild-type and *Slc29a1*-null mice, suggesting that this effect is independent of the actions of NBMPR as an ENT1 blocker. The concentration NBMPR used in this experiment (10 μM, to ensure complete ENT1 block) was >1,000-fold higher than its K_i_ for ENT1 inhibition [52]. It is possible that NBMPR at these high concentrations is having nonspecific (and undocumented) actions on other components of the α-adrenergic signalling system.

### Effect of adenosine

Adenosine was unable to relax pre-constricted aortas from wild-type mice, but did have a significant relaxant effect on the aortas from *Slc29a1*-null mice. This is likely due to a longer half-life of the adenosine in the extracellular media in the absence of ENT1-mediated uptake. The fact that SCH58261 blocked this effect of adenosine confirmed that it was due to interactions with extracellular adenosine A2 receptors. It is also noteworthy that SCH58261 on its own led to an increase in contraction of the aortas, with a greater effect apparent in the tissue from *Slc29a1*-null mice. This supports the concept that there in an enhanced adenosine-mediated vasodilatory `tone`in *Slc29a1*-null mice, with the aortic rings from these mice having an increased adenosine A2 receptor stimulation due to higher extracellular adenosine concentrations. It should be noted that the concentrations of adenosine required to cause vasodilation of the aortic rings, even in the absence of functional ENT1 (>100 μM), are an order of magnitude higher than levels normally achieved *in vivo*. A similar low sensitivity of mouse aorta to the vasodepressant effects of adenosine has been described by others [34, 53]. Therefore, although adenosine A2a receptors are expressed in the mouse thoracic aorta and can mediate adenosine-induced vasodilation, the vasodilatory activity of adenosine is not likely to be physiologically relevant in the mouse thoracic aorta.

### Effect of hypoxia

Prolonged hypoxia, as employed in this study, has been shown to impair vascular reactivity to phenylephrine in rat aorta [54, 55]. Our data showed a similar effect of hypoxia on vascular reactivity in mouse aorta. Hypoxia is known to enhance the extracellular levels of adenosine in vascular tissue, which leads to increased vasodilation and protection of vascular endothelial cells from the hypoxic insult by promoting vascular barrier integrity and reducing tissue accumulation of neutrophils [56–58]. This effect of hypoxia on vascular adenosine levels is due, in part, to a HIF-1α-dependent transcriptional repression of ENT1 [58, 59]. A similar mechanism has also been described for enhancement of extracellular adenosine in response to inflammatory lung injury, where both ENT1 and another member of the equilibrative nucleoside transporter family, ENT2, were repressed in this model [60]. HIF-1α-mediated repression of ENT2 has also been implicated in adenosine-mediated attenuation of hypoxia-associated inflammation of intestinal epithelium [61].

Under physiological conditions, most of the adenosine comes from extracellular sources. However, in times of cellular metabolic stress, such as that caused by hypoxia, adenosine is formed from the metabolism of intracellular ATP and is released from cells to mediate its cardioprotective effects via interactions with adenosine receptors on the cell membranes. Since ENT1 is the primary mechanism by which adenosine is released from cells, we hypothesized that the protective effect of adenosine under hypoxic conditions would be reduced in the ENT1-deficient aortas from the *Slc29a1*-null mice. Hypoxia had no significant effect on the phenylephrine-induced contraction of aortas from wild-type mice, suggesting the activity of a robust endogenous protective mechanism. However, in the aortas from *Slc29a1*-null mice, hypoxia caused a significant reduction in the maximum contraction induced by phenylephrine, which supports the hypothesis that adenosine release is needed for its protective effects under these conditions. To confirm that this difference was due to the inability of the aortas from *Slc29a1*-null mice to release adenosine, we conducted a similar study after pre-incubation of the aortas of wild-type and *Slc29a1*-null mice with the ENT1 inhibitor NBMPR. As would be expected, NBMPR had no significant effect on the phenylephrine-induced tone in the ENT1-deficient aortas from the *Slc29a1*-null mice. However, in contrast to expectations, NBMPR enhanced the maximum phenylephrine induced contraction in the aortas from wild-type mice under hypoxic conditions, opposite to that seen using aortas from *Slc29a1*-null mice. This may reflect a nonspecific action of NBMPR on the tissue, not related to its ENT1-blocking activity, similar to that invoked for its effect on phenylephrine potency in aortas from *Slc29a1*-null mice. Alternatively, NBMPR could be preventing adenosine from being released from cells during hypoxia and thereby maintaining intracellular ATP stores which would be beneficial in the maintenance of contractile function in the wild-type mice. However, the latter scenario is not compatible with our findings that aortas from *Slc29a1*-null mice have reduced functionality relative to the aortas from wild-type mice under hypoxic conditions. An alternate explanation is that exposure of the aortas from wild-type mice to NBMPR prior to hypoxia may induce a preconditioning-like effect on the tissue via enhancement of extracellular adenosine levels. To test this hypothesis, we exposed aortas from wild-type and *Slc29a1*-null mice to adenosine for 30 min (same protocol as used with NBMPR) prior to assessing the ability of phenylephrine to constrict the tissue under hypoxic conditions. As was seen using NBMPR, pre-treatment of aortas from wild-type mice with adenosine enhanced their response to phenylephrine, but this was not seen using aortas from *Slc29a1*-null mice. The similarity in the effects of the NBMPR and adenosine pre-treatment on the maximum contraction generated by phenylephrine in the aortas from wild-type mice suggests that NBMPR was enhancing extracellular adenosine levels and thereby mediating an adenosine-induced preconditioning-like effect. Others have also shown that treatment of tissues with adenosine [3], or with the adenosine uptake inhibitor dipyridamole [62], can protect those tissues from subsequent ischemia-reperfusion induced injury. Given that aortas from the *Slc29a1*-null mice have been chronically exposed to elevated adenosine, they may already be preconditioned and consequently further exposure to adenosine or NBMPR has no effect. In support of this conjecture, it has been noted that cardiomyocytes isolated from *Slc29a1*-null mice had increased expression of HIF-1α under normoxic conditions which mimicked the response of wild-type cardiomyocytes in response to hypoxic challenge, and that hypoxia had no effect on HIF-1α in the *Slc29a1*-null cells [44]. Isolated hearts and livers from the *Slc29a1*-null mice were also shown to be relatively resistant to ischemia-reperfusion injury compared with the tissues from wild-type mice [16, 24], and those investigators suggested that this was due to a preconditioned phenotype of the *Slc29a1*-null mice. These data support a role for ENT1 in the regulation of the protective effects of adenosine on contractile function in elastic conduit arteries such as thoracic aorta. This study also suggests that thoracic aorta can be preconditioned by exposure to relatively high concentrations of adenosine, thereby limiting the dysfunction caused by hypoxia. Suppression of ENT1 function may thus be a viable therapeutic option to pursue for the treatment of conditions involving aortic dysfunction.

## Supporting information

S1 Table qPCR primer sequences. The forward (Fwd) and reverse (Rev) primers used for qPCR measurement of the indicated gene transcripts are shown along with the optimum annealing temperature.
(DOCX)
