# Supplementary material for: Changes in aortic reactivity associated with the loss of equilibrative nucleoside transporter 1 (ENT1) in mice
Source: PLoS One. 2018 Nov 8;13(11):e0207198. doi: 10.1371/journal.pone.0207198 (PMC6224178; doi:10.1371/journal.pone.0207198)
Supplement: S1 Table — The forward (Fwd) and reverse (Rev) primers used for qPCR measurement of the indicated gene transcripts are shown along with the optimum annealing temperature. (DOCX) [file pone.0207198.s001.docx]

**S1 Table:** **qPCR Primer Sequences**

| Gene | Primer Sequence (5’ to 3’) | Temperature (^o^C) |
| --- | --- | --- |
| *Ada* | Fwd – ACAATCAGAAGACCGTGGTGGCTA  Rev – TCTTTACTGCGCCCTCATAGGCTT | 60 |
| *Adk* | Fwd – CTCAGTGGTTGATTCAGGAGC  Rev – CAGCCTTACGCTTCAGGATCT | 60 |
| *Adora1* | Fwd – ATCCCTCTCCGGTACAAGACAGT  Rev – ACTCAGGTTGTTCCAGCCAAAC | 56 |
| *Adora2a* | Fwd – CCGAATTCCACTCCGGTACA  Rev – CAGTTGTTCCAGCCCAGCAT | 56 |
| *Adora2b* | Fwd – ATGCAGCTAGAGACGCAAGAC  Rev – GGGATACCAGAAAGTAGTTGGTG | 60 |
| *Adora3* | Fwd – ACTTCTATGCCTGCCTTTTCATGT  Rev – AACCGTTCTATATCTGACTGTCAGCTT | 56 |
| *Enpp1* | Fwd – CAAAACCCACAATGACTGTTGC  Rev – CTGGACACTCCGGGGTATCG | 56 |
| *Slc29a1* | Fwd – CAAGTATTTCACAAACCGCCTGGAC  Rev – GAAACGAGTTGAGGCAGGTGAAGAC | 56 |
| *Slc29a2* | Fwd – TCATTACCGCCATCCCGTACT  Rev – CCCAGTTGTTGAAGTTGAAAGTG | 56 |
| *Slc29a3* | Fwd – AGCCTCACTCTTTCCAGCTGACAT  Rev – ACTTGGTGTCTGTGTCGTTCCTGT | 60 |
| *Slc29a4* | Fwd – TGCTGTCCATTGCGGTAACCTACT  Rev – TTGCCCACAAAGTCTGACAGGTTG | 56 |
| *Nt5e* | Fwd - GGAGCTCACGATCCTGCACACAAA  Rev – TCCCAGTGCCATAGCATCGTAGCC | 56 |
| *Pnp* | Fwd - GCCGACTGGTGTTTGGATTGCTG  Rev – TCGTTGCTCCCCCATTTGTTTCC | 56 |
| *Ubc* | Fwd - GACCAGCAGAGGCTGATCTT  Rev - CCTCTGAGGCGAAGGACTAA | 56 |
| *ActB* | Fwd - ACTGTCGAGTCGCGTCCA  Rev - CGTCATCCATGGCGAACT | 56 |
| *eIF4a2* | Fwd - TCGAAATGAAATGCAGAAGTTG  Rev - GCTTCGTCCAAAACGAACAT | 53 |
| *Atp5b* | Fwd – TCCAGCAGATTTTAGCAGGTG  Rev - GCTGGAGTCCCTCACGAC | 56 |
